# A dose-finding study of carboplatin–epirubicin–docetaxel in advanced epithelial ovarian cancer

**DOI:** 10.1038/sj.bjc.6600259

**Published:** 2002-05-06

**Authors:** V J O'Neill, S B Kaye, N S Reed, J Paul, J A Davis, P A Vasey

**Affiliations:** Cancer Research UK Department of Medical Oncology, Beatson Oncology Centre, Western Infirmary, Glasgow GL1 6NT, UK; Department of Gynaecology, Stobhill General Hospital, Glasgow, UK

**Keywords:** docetaxel, epirubicin, carboplatin, advanced epithelial ovarian cancer, dose-finding study

## Abstract

The docetaxel–carboplatin combination is active and well tolerated in patients with epithelial ovarian cancer. We added epirubicin to this combination to investigate additional benefits of anthracyclines in epithelial ovarian cancer. Twenty-one patients, FIGO Ic-IV, performance status 0–1, were treated in four dose cohorts. Docetaxel was fixed at 75 mg m^−2^, carboplatin doses were AUC 4–5 and epirubicin doses were 50–60 mg m^−2^. Drugs were given on day 1, every 3 weeks, except in cohort 3, where epirubicin was given on day 8. Dexamethasone was given prophylactically. One dose-limiting toxicity occurred in cohorts 1, 2 and 4, two occurred in cohort 3. Complicated neutropenia occurred in two patients in cohorts 1 and 2 and one patient in cohorts 3 and 4. Two patients experienced grade III diarrhoea or stomatitis in cohort 1 and two in cohort 3. There were no treatment-related deaths. Grade II sensory neuropathy occurred in one patient. No cardiac toxicity or significant oedema was observed. The overall response rate was 36%, and 62% were CA125 responders. The predefined maximum tolerated dose was exceeded in cohort 3. The cohort 4 dose level (epirubicin 50 mg m^−2^, carboplatin AUC 4, docetaxel 75 mg m^−2^), warrants further study.

*British Journal of Cancer* (2002) **86**, 1385–1390. DOI: 10.1038/sj/bjc/6600259 www.bjcancer.com

© 2002 Cancer Research UK

## 

Cancer of the ovary is the fourth most common cause of cancer death in women, and is the leading cause of gynaecological cancer death in the developed world (Boring *et al*, 1992). Around 90% of cases are epithelial carcinomas, and approximately 75% will have spread beyond the ovaries at the time of diagnosis (Young *et al*, 1993). Because of this advanced presentation, only a minority of women will have surgically curable localised disease, and consequently systemic chemotherapy has become the mainstay of treatment. The high response rates observed with chemotherapy have not readily translated into major long-term survival gains, and the overall 5-year survival is still less than 30% (Neijt *et al*, 1991). The need for new therapeutic strategies is therefore clear.

Combination chemotherapy with cisplatin has achieved higher response rates and improved median survival in randomised trials (Advanced Ovarian Cancer Trialist Group, 1991), and combination chemotherapy with cisplatin and an alkylating agent remained standard treatment for advanced ovarian cancer until recently. In 1996, a randomised trial from the Gynaecologic Oncology Group (GOG) demonstrated clear superiority of cisplatin combined with paclitaxel (McGuire *et al*, 1996), a result confirmed in the European Intergroup study (Piccart *et al*, 2000). Paclitaxel–cisplatin was rapidly adopted worldwide as the standard of care in this setting. Interestingly, GOG 132 (Muggia *et al*, 2000) showed no survival advantage for paclitaxel–cisplatin over either drug given as monotherapy. One interpretation of this trial is that patients who did not receive paclitaxel during the study may have received it following disease progression, thus essentially having sequential treatment. Therefore, the sequential administration of these agents may be as efficacious as concomitant combination, although the trial was not specifically designed to examine this hypothesis.

Substitution of carboplatin for cisplatin in combination with paclitaxel has resulted in a reduced incidence of emesis and neurotoxicity, albeit at the expense of greater myelosuppression, and is supported by data from three randomised studies (Neijt *et al*, 1997; Ozols *et al*, 1999; du Bois *et al*, 1999). Results from these trials justify the choice of carboplatin–paclitaxel as preferred first-line treatment, with equivalent efficacy and superior tolerability compared with cisplatin–paclitaxel.

Docetaxel (Taxotere®) has demonstrated substantial single-agent efficacy that is at least comparable to paclitaxel in advanced, platinum-refractory ovarian cancer (Kaye *et al*, 1997) and has also been combined with cisplatin in a multicentre pilot/feasibility study (Vasey *et al*, 1999). The feasibility of a carboplatin–docetaxel combination as first-line chemotherapy for patients with advanced epithelial ovarian cancer has been explored in a large, multicentre dose-finding study (Vasey *et al*, 2001a). Here, significant neurotoxicity was unusual, although myelosuppression was common. Despite this, sepsis was rare and neither prophylactic antibiotics nor growth factors were routinely necessary. The overall response rate was 66%, although 75% of patients fulfilled CA125 response criteria. Carboplatin AUC 5 and docetaxel 75 mg m^−2^ were considered worthy of further investigation. Carboplatin AUC 5 and docetaxel 75 mg m^−2^ have been used in a randomised phase III study comparing docetaxel–carboplatin with paclitaxel–carboplatin (Vasey *et al*, 2001b). Preliminary results suggest that these two treatment arms have comparable efficacy, with a significantly lower incidence of neurotoxicity in the docetaxel–carboplatin arm *vs* paclitaxel–carboplatin, providing additional evidence for the activity and tolerability of this combination as first-line treatment of ovarian cancer. The combination of docetaxel and carboplatin has also been successfully administered to patients with ovarian, peritoneal and fallopian tube malignancies (Markman *et al*, 2001).

There is evidence for additional benefit when incorporating an anthracycline into combination chemotherapy regimens for advanced ovarian cancer. Four randomised trials have compared an anthracycline-containing regimen – cyclophosphamide, adriamycin and cisplatin (CAP) – with a non-anthracycline containing regimen – cisplatin and cyclophosphamide (CP) (Conte *et al*, 1986; Bertelsen *et al*, 1987; Omura *et al*, 1989; GICOG, 1992). All of these trials demonstrated a slight non-significant trend towards a survival advantage for CAP. Furthermore, a published overview of two large meta-analyses, using individualised data from over 1700 untreated patients, demonstrated that the addition of anthracycline significantly improved survival (HR 0.85, *P*=0.03) (A'Hern and Gore, 1995). The most commonly used anthracycline is doxorubicin; however, epiruibicin is known to have essentially the same spectrum of activity, with less cardiotoxicity, and therefore a more favourable toxicity profile. In addition, epirubicin 60 mg m^−2^ has been added to the combination of paclitaxel 175 mg m^−2^ and carboplatin AUC 7, with a high response rate and manageable toxicities (Hill *et al*, 1997).

In this study, we added epirubicin to the docetaxel plus carboplatin combination in order to determine the feasibility and safety of the combination in patients with advanced ovarian cancer.

## MATERIALS AND METHODS

### Patients

Eligible women had histologically verified epithelial ovarian cancer, were over 18 years old, and had FIGO stages Ic–IV with or without successful cytoreductive surgery at staging laparotomy. Stage Ic disease was limited to patients with malignant cells in ascitic fluid or peritoneal washings, pre-operative capsular rupture or surface tumour. Stage Ic patients with intra-operative ruptured capsule only were ineligible. Patients were required to have an ECOG performance status of ⩽2, and adequate bone marrow and hepatic function as evidenced by: neutrophils ⩾1. 5×10^9^ l^−1^, platelets ⩾100×10^9^ l^−1^, bilirubin<upper limit of normal (ULN), aminotransferases AST/ALT <1.5×LTLN, alkaline phosphatase (ALP) <3×ULN. Adequate renal function was required and defined by serum creatinine <1.25×ULN. Written, informed consent, in compliance with the recommendations of the Declaration of Helsinki, was obtained in all cases.

Patients were ineligible for study entry if they had received prior treatment with chemotherapy or radiotherapy, or had any prior malignancy (except for curatively treated carcinoma *in situ* of the uterine cervix or basal cell carcinoma of the skin). Patients with borderline ovarian tumours or abdominal adenocarcinoma of unknown origin were excluded, as were those with clinically significant pleural effusions or ascites unless confirmed cytologically to be due to ovarian cancer. Patients were also ineligible if there was a history of medically significant atrial or ventricular dysrrhythmias, congestive heart failure, angina pectoris or documented myocardial infarction within the 6 months preceding study entry. A pretreatment electrocardiogram (ECG) showing evidence of infarction with no other clinical features did not preclude study entry. Additional contraindications included: active infection or serious intercurrent illness that was judged by the investigators likely to impair the patient's ability to receive protocol therapy; a history of prior serious allergic reactions; and symptomatic peripheral neuropathy>grade II. Pregnant or lactating women were ineligible, but potentially fertile women using adequate contraception were allowed treatment. A diagnosis of insulin-dependent diabetes mellitus or other relative contraindications to corticosteroid administration were discussed with the investigators prior to enrolment.

### Treatment plan and administration

Epirubicin 50–60 mg m^−2^, docetaxel 75 mg m^−2^ and carboplatin AUC 4–5 were administered consecutively on day 1 of a 21-day cycle in three of four treatment cohorts. In cohort 3, epirubicin 50 mg m^−2^ was administered on day 8 (Table 1

**Table 1:**
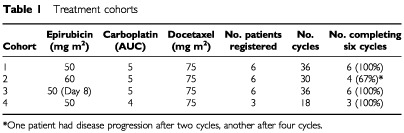
Treatment cohorts

). The anticipated total number of cycles was six. Carboplatin dosing was determined prior to the first cycle by the formula (glomerular filtration rate (GFR) + 25)×desired AUC (Calvert *et al*, 1989), where the GFR was measured by ^51^Cr EDTA (Chantler *et al*, 1969). This dose remained fixed throughout subsequent cycles, unless de-escalation was required as a result of toxicity. Patients who had either a partial response or stable disease after six cycles were allowed to receive further chemotherapy with three cycles of single-agent carboplatin, AUC 5–7, depending on the clinician's preference. On the completion of first-line chemotherapy, patients ceased all cytotoxic chemotherapy until documented clinical progression. The appropriateness of either second-look or interval cytoreductive surgery was determined on an individual patient basis, as this was not a protocol requirement. Six patients were entered into each dose cohort until they had completed two full treatment cycles. Escalation to the next dose cohort proceeded if fewer than two of the six patients developed dose-limiting toxicity (DLT) during Cycles 1 or 2. Dose-limiting toxicity was defined as any of the following toxicities occurring in the first two cycles of treatment: (a) complicated or prolonged grade IV neutropenia; (b) complicated grade IV thrombocytopenia and/or a need for platelet transfusion; (c) any grade III non-haematological toxicity excluding emesis and alopecia. The maximum tolerated dose (MTD) was defined as the dose level at which two or more of the first six patients followed up for two complete cycles experienced a DLT.

Premedication consisted of oral dexamethasone, 8 mg bid for 3 days, starting the day before chemotherapy. Epirubicin was administered over approximately 15 min into the side port of a fast-flowing drip. Docetaxel was reconstituted in 250 ml of 5% glucose solution and administered by intravenous infusion over 60 min. Carboplatin was then administered in 500 ml of 5% glucose solution over 30–60 min. Prophylactic intravenous antiemetics (8 mg dexamethasone plus either 3 mg granisetron or 8 mg ondansetron) were administered to all patients, and were timed to be given immediately prior to the epirubicin injection. All patients were routinely prescribed oral domperidone 20 mg tid or qid as required, for 5–7 days following chemotherapy.

### Dose and schedule modifications

Treatment was administered on day 1 of each planned 21-day cycle if the patient's neutrophil count was >1.5×10^9^ l^−1^ and platelet count was >100×10^9^ l^−1^; values less than this necessitated a treatment delay until recovery. In cohort 3, epirubicin was administered on day 8, irrespective of full blood count. Any delay of more than 2 weeks for haematological recovery necessitated termination of protocol therapy. Dose reductions were based upon nadir blood counts. Any grade IV neutropenia that lasted at least 7 days and/or was complicated by fever resulted in a reduction of docetaxel dose by 15 mg m^−2^ on all subsequent cycles. Any such neutropenic events were treated at the time with antibiotics, and granulocyte colony stimulating factor (G-CSF) was added if considered appropriate by the investigator. The occurrence of neutropenic fever also resulted in prophylactic oral antibiotics (ciprofloxacin 250 mg bid days 5–15) being prescribed for each subsequent treatment cycle. If complicated or prolonged neutropenia occurred again, despite dose reductions and prophylactic antibiotics, subsequent cycles were delivered with subcutaneous G-CSF 300 μg day^−1^ from days 5–14 or until the neutrophil count was >1.0 10^9^ l^−1^ and rising. If this was considered inappropriate, the remaining cycles of chemotherapy were administered without epirubicin, using a dose of docetaxel 75 mg m^−2^ and carboplatin AUC 5. Grade IV thrombocytopenia requiring platelet transfusion and/or complicated by haemorrhage resulted in a reduction of the carboplatin dose by 10% in all subsequent cycles.

Abnormalities of hepatic function, as evidenced by AST/ALT and/or ALP elevations to>grade I during treatment, resulted in the patient's withdrawal from protocol therapy and continued treatment with carboplatin as a single agent.

Treatment delays were planned for patients who developed severe skin toxicity (>grade II) for a maximum of 2 weeks until recovery to <grade I, when they could be re-treated with a 10–15 mg m^−2^ reduction of docetaxel. Mucositis >grade II necessitated a treatment delay of maximum 2 weeks until resolution of lesions, and a subsequent docetaxel dose reduction as above. No dose reductions were planned on the basis of docetaxel-induced fluid retention. The development of grade III/IV neurotoxicity – motor, sensory or otological – necessitated termination of protocol therapy.

Mild hypersensitivity reactions were treated by slowing down the docetaxel infusion. Severe hypersensitivity reactions were terminated with appropriate drug therapy (adrenaline, antihistamines or corticosteroids, depending upon the severity of the reaction). Re-challenge after recovery from a hypersensitivity reaction was allowed if clinically indicated, and was generally done within 3 h. Later re-challenges (within 3–24 h) were required to be further premedicated with high-dose dexamethasone and chlorpheniramine. Further hypersensitivity reactions necessitated withdrawal from study.

### Patient evaluation and clinical assessments

Patients underwent full physical examination, including vaginal/rectal examination. Baseline investigations prior to study entry included: full blood count and differential white cell count, biochemical profile (including urea, creatinine, sodium, potassium, calcium, magnesium, AST, ALT, ALP, bilirubin, total protein, albumen, ^51^Cr EDTA measurement of GFR, chest X-ray, glucose), CA125 and 12-lead ECG. The size and extent of residual disease were documented by computed tomography (CT) scan of abdomen and pelvis. Patients' weight and ECOG performance status were noted at baseline.

During chemotherapy, patients were seen weekly for full blood count, serum chemistry and documentation of treatment-related toxicity using the National Cancer Institute of Canada Expanded Common Toxicity Criteria (NCIC-CTC, version 2.0). Prior to each treatment cycle, patients were weighed and had a full physical examination plus CA125 estimations. Response to chemotherapy was assessed after three and six (and, if appropriate, nine) courses of chemotherapy by the same imaging technique used at baseline. Clinical and radiological tumour response were graded according to standard criteria (Miller *et al*, 1981); CA125 responses were graded according to the schema from Rustin and co-workers (Rustin *et al*, 1996).

Following completion of protocol chemotherapy, patients were followed-up 2-monthly for the first 2 years, 4-monthly to 5 years and annually thereafter. Pelvic examination was carried out at each follow-up visit, along with CA125 measurement. CT scans were carried out if progressive disease was suspected clinically or CA125 levels began to increase.

### Statistical methods

Analysis of variance (ANOVA) techniques were used to compare cycle 1 nadir neutrophil and platelet counts between cohorts (after suitable transformations to make the data approximately normal). Cumulative haematological toxicity was examined using repeated measures ANOVA. Proportions were compared using Pearson's chi-square test (unadjusted). All survival times were dated from when the patient was registered on the study. Progression-free survival was the time from registration to progression or death, from any cause. Survival curves were determined using Kaplan–Meier estimates.

## RESULTS

Twenty-one patients treated at the Beatson Oncology Centre, Glasgow, were enrolled in this study. In total, 120 cycles of docetaxel–carboplatin–epirubicin chemotherapy were administered to these patients over four dose cohorts (Table 1). Pretreatment patient characteristics are shown in Table 2

**Table 2:**
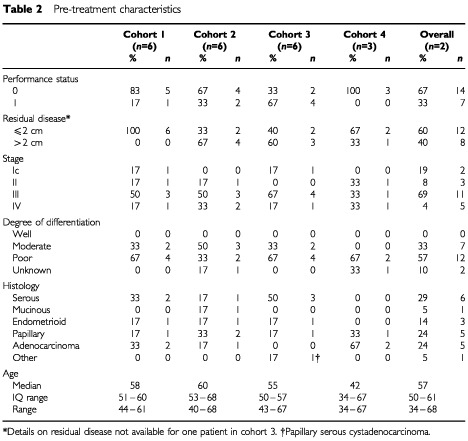
Pre-treatment characteristics

. All patients had epithelial ovarian adenocarcinoma: serous/papillary adenocarcinoma 11 (52%); endometrioid 3 (14%); mucinous 1 (5%); other 1 (5%). The majority of patients (12 patients; 57%) had poorly differentiated tumours. The median age was 57 years (range 34–68 years). Sixteen patients (73%) were FIGO stage III or IV at presentation, and all patients had a performance status of 0–1. The majority of patients (12 patients; 57%) had been optimally debulked prior to treatment.

### Toxicity

A total of 19 patients (90%) received the planned six cycles of treatment, and there were no withdrawals from the study because of toxicity. Haematological toxicity was seen in all treatment cohorts and is presented in Table 3

**Table 3:**
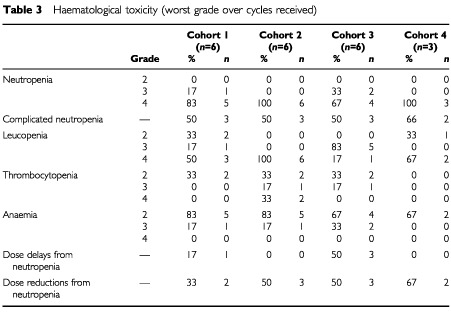
Haematological toxicity (worst grade over cycles received)

. Grade IV neutropenia was observed in 18 patients (86%), with nine patients (43%) experiencing prolonged (>5 days) neutropenia and a further five patients (24%) experiencing grade IV neutropenia with fever. In total, 11 patients had complicated or prolonged neutropenia. However, only one patient had grade IV neutropenia with pyrexia lasting more than 2 days, and there were no sepsis-related deaths. Four patients (19%) required a dose delay secondary to neutropenia, one in cohort 1 and three in cohort 3, and 10 patients (48%) required dose reductions. Two patients, both in cohort 2, experienced grade IV thrombocytopenia, but there were neither episodes of haemorrhage nor any requirement for prophylactic platelet transfusion. Significant anaemia was not observed: while almost all patients (20 patients, 95%) experienced anaemia at grade II or III, no patient had grade IV anaemia. Only one cycle of chemotherapy (Cycle 5, cohort 1) was delayed due to the need for blood transfusion.

Significant non-haematological toxicity was unusual, and treatment was generally well tolerated by patients. The most common toxicities were grade II stomatitis (10 patients; 47%) and, predictably, grade III alopecia (14 patients; 67%). In addition, nine patients (43%) experienced grade II lethargy. Table 4

**Table 4:**
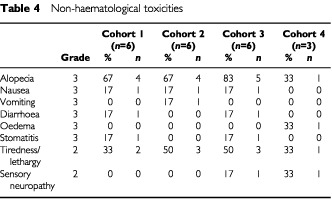
Non-haematological toxicities

lists all significant non-haematological toxicities. There were no reports of grade IV nausea or emesis. One patient reported grade III vomiting and three reported grade III nausea. Grade III diarrhoea was experienced by two patients (10%) and two patients experienced grade III mucositis.

Severe neurotoxicity was not encountered. Two patients (10%) experienced grade II sensory neuropathy, which had fully resolved by 7 months. No motor neuropathy was identified. Two patients had grade II peripheral oedema, while one patient had grade III oedema requiring treatment with diuretics. One patient experienced grade III polyarthropathy that had resolved at most recent follow-up (22 months). No clinically evident cardiac toxicity was observed. There was one incidence of grade II hypersensitivity reaction, which took the form of generalised urticaria. After stopping the infusion, the patient was re-challenged with no further problems.

In summary, the maximum tolerated dose was exceeded in cohort 3. Overall, there was one DLT in cohort 1, one in cohort 2, two in cohort 3 and one in cohort 4. In addition, a further two patients experienced complicated neutropenia in cohort 1, two in cohort 2, one in cohort 3 and one in cohort 4 (Table 5

**Table 5:**
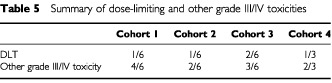
Summary of dose-limiting and other grade III/IV toxicities

). Two patients experienced grade 3 diarrhoea or stomatitis in cohort 1 and two in cohort 3. There were no treatment-related deaths.

### Response and survival

Eleven patients (52%) had radiologically evaluable disease at outset: four patients (19%) had a complete response, three patients (14%) had stable disease and four (19%) had progression of their disease. The overall radiological response rate is therefore 36%. Sixteen patients were evaluable for CA125 response, 10 of whom satisfied strict Ruskin criteria to qualify as responders (62%). The median follow-up for living patients is 20 months (range 17–22 months). The median progression-free survival time is 12 months (95% CI=6–18 months) and the 1-year survival rate is 62% (SE=11%).

## DISCUSSION

We report here the first experience of carboplatin, epirubicin and docetaxel in combination as first-line treatment for advanced epithelial ovarian cancer. The theoretical basis for this combination is clear, since these three classes of drugs are non-cross-resistant and show significant activity in ovarian cancer. In addition, there are *in vitro* data suggesting that synergy exists between taxanes and anthracyclines (Bruckner *et al*, 1994).

As anticipated, the myelosuppressive capacity of this regimen was high but nevertheless manageable. Overall, 86% of patients experienced grade IV neutropenia, somewhat less than that reported for the combination of paclitaxel, anthracycline and platinum treatment (i.e. 100%) in a similar patient population (Gregory *et al*, 2000). However, 11 patients (51%) experienced complicated or prolonged neutropenia. The incidence of thrombocytopenia in our patients was less than in the study cited (18 *vs* 50%). The fact that neutropenia was not accompanied by sepsis in the majority (81%) of cases is probably attributable to the low incidence of grade III/IV mucositis or diarrhoea. Nevertheless, grade III and IV toxicities occurred in cohorts 1–3 and dose reductions and delays were seen as a consequence. Administration of epirubicin on day 8 in cohort 3 was done in an attempt to reduce the incidence of grade IV neutropenia, albeit at the expense of an additional day's visit to the clinic. Dose limiting toxicities were however still observed. The dose level used in cohort 4 – namely epirubicin 50 mg m^−2^, carboplatin AUC 4 and docetaxel 75 mg m^−2^ – is in theory worthy of further study. However, a carboplatin dose of AUC 4 (albeit calculated via measured GFR) may be considered suboptimal therapy, even in combination treatment. For this reason, cohort 4 was stopped after the accrual of three patients. Routine use of G-CSF support has been shown to substantially reduce myelosuppression in high-dose treatment of breast cancer patients receiving 250 mg m^−2^ paclitaxel, 90 mg m^−2^ doxorubicin and 3 mg m^−2^ cyclophosphamide (Hudis *et al*, 1995). One option would therefore be to include G-CSF in the schedule. An alternative would be to substitute pegylated doxorubicin (Muggia *et al*, 1997) for epirubicin, since this might be expected to result in a reduced rate of myelosuppression, albeit at the expense of other non-haematological toxicities such as hand–foot syndrome.

Neurotoxicity was not a significant problem with this regimen, which confirms data for docetaxel–carboplatin alone. Only two patients (10%) reported mild sensory neuropathy, which fully resolved over several months, in concordance with the reported low incidence of neurotoxicity for single-agent docetaxel. The striking lack of neurotoxicity with this regimen is in contrast to the significant neurotoxicity of paclitaxel and paclitaxel-containing combinations, and provides a cogent argument for making docetaxel the taxane of choice in this setting. Survival comparisons of docetaxel and paclitaxel plus carboplatin are awaited – in particular, mature data from the SCOTROC trial (Scottish Randomised Trial in Ovarian Cancer). Preliminary results from this study (Vasey, 2001b), in which the combination of docetaxel–carboplatin was compared with paclitaxel–carboplatin, suggest that the former combination is significantly less neurotoxic than paclitaxel–carboplatin (sensory neuropathy occurred in 11% and 30% of patients, respectively; *P*<0.001). Docetaxel-related peripheral oedema was mostly preventable with steroid pre-medication, and only one patient experienced a moderate hypersensitivity reaction to the drug.

Cardiac function was not measured objectively in this study, since it was felt that the incidence of cardiotoxicity in anthracycline-naïve patients, particularly with the use of epirubicin, was likely to be very low. Consequently, careful clinical assessment failed to reveal any development of cardiac dysfunction during the study.

Objective response rates in this trial were less than might have been expected, and less than in other published series of anthracycline–platinum–taxane treated patients (Gregory *et al*, 2000). For example, in the SCOTROC study, both objective and CA125 response rates were similar in patients treated with either docetaxel–carboplatin or paclitaxel–carboplatin (Vasey, 2001b). The lower response rates observed in our study are likely to be a consequence of both the small patient sample and possibly a combination of the frequent dose delays and reductions required in response to toxicity, as 52% of patients required a dose reduction during treatment. It is worth noting that only 50% of patients in the present study had radiologically evaluable disease and therefore comparison of response rates with other studies is inappropriate. The CA125 response rate (62%) is more in line with expected response rates. However, this trial was initiated as a toxicity/feasibility study and comparisons of efficacy between phase I and II studies are not informative.

In spite of great progress in the treatment of ovarian cancer, several questions remain unanswered. One concerns the best taxoid to use, balancing activity and tolerability profiles. Considerable evidence now supports the use of docetaxel and carboplatin in patients with advanced ovarian cancer, in particular the reduced incidence of neurotoxicity in docetaxel-containing combinations, compared with those containing paclitaxel. The optimum administration schedule is also under active debate, in particular the scheduling of cytotoxics. This might take the form of examining alternating doublets, as in GOG 182. In this five arm, prospective, randomised trial, which aims to accrue over 5000 patients, four cycles of gemcitabine–carboplatin followed by four cycles of paclitaxel–carboplatin will be compared with four cycles of liposomal doxorubicin–carboplatin followed by four cycles of paclitaxel–carboplatin, four cycles of topotecan–carboplatin followed by four cycles of paclitaxel–carboplatin, eight cycles of gemcitabine–paclitacel–carboplatin and eight cycles of paclitaxel–carboplatin. Substitution of pegylated doxorubicin for epirubicin, as in the above study, could be expected to produce less myelosuppression and therefore fewer dose reductions and delays. An alternative approach is to utilise sequential scheduling, whereby the new agent is delivered either alone or in combination after initial treatment with carboplatin. Sequential chemotherapy has become firmly established in the treatment of breast cancer (Bonadonna *et al*, 1995). The feasibility of this approach is also being examined in the SCOTROC-2 study programme, which is currently recruiting patients in the UK and Europe. This randomised Phase III trial is comparing four cycles of single-agent carboplatin followed by four cycles of docetaxel alone or in combination with CPT-11 or gemcitabine. Results are expected in the first quarter of 2002.

In conclusion, the combination of carboplatin–epirubicin–docetaxel shows activity in patients with ovarian cancer, but toxicity-induced dose reductions and delays may limit its utility. There were no patient withdrawals as a result of toxicity, and the striking lack of neurotoxicity in this study is in line with docetaxel–carboplatin administration. The recently reported AGO–GINECO trial, examining the use of paclitaxel-carboplatin with or without epirubicin, has to date failed to show any significant advantage for the triple combination (all given on day 1), although follow-up is short (du Bois *et al*, 2001). Therefore, further studies should examine the role of scheduling in the activity and tolerability of these agents.

## References

[bib1] Advanced Ovarian Cancer Trialist Group1991 Chemotherapy in advanced ovarian cancer: an overview of randomized clinical trials Br Med J 303 884893 1834291 10.1136/bmj.303.6807.884 PMC1671193

[bib2] A'HernRPGoreME1995 Impact of doxorubicin on survival in advanced ovarian cancer J Clin Oncol 13 726732 7884432 10.1200/JCO.1995.13.3.726

[bib3] BertelsenKJakobsenAAndersenJEet al1987 A randomized study of cyclophosphamide and cis-platinum with or without doxorubicin in advanced ovarian carcinoma Gynecol Oncol 28 161169 3311924 10.1016/0090-8258(87)90210-1

[bib4] BonadonnaGZambettiMValagussaP1995 Sequential or alternating doxorubicin and CMF regimens in breast cancer with more than three positive nodes. Ten-year results JAMA 273 542547 7837388

[bib5] BoringCCSquiresTSTongT1992 Cancer statistics CA 42 19381728335

[bib6] BrucknerHWCagnoniPJLeeJMet al1994 A sequence of adriamycin and Taxol® infusions for refractory ovarian cancer Proc Am Soc Clin Oncol 13 276

[bib7] CalvertAHNewallDRGumbrellLAet al1989 Carboplatin dosage: prospective evaluation of a simple formula based on renal function J Clin Oncol 7 17481756 2681557 10.1200/JCO.1989.7.11.1748

[bib8] ChantlerCGarnettESParsonsVet al1969 Glomerular filtration rate measurement in man by the single injection method using ^51^CrEDTA J Clin Sci 37 169190 4980763

[bib9] ContePFBruzzoneMChiaraSet al1986 A randomized trial comparing cisplatin plus cyclophosphamide versus cisplatin, doxorubicin, and cyclophosphamide in advanced ovarian cancer J Clin Oncol 4 965971 3519886 10.1200/JCO.1986.4.6.965

[bib10] du BoisALueckHJMeierWet al1999 Cisplatin/paclitaxel vs carboplatin/paclitaxel in ovarian cancer: update of an Arbeitsgemeinschaft Gynaekologische Onkologie (AGO) study group trial Proc Am Soc Clin Oncol 18 1374[Abstr]

[bib11] du BoisAWeberBPfistererJGoupilAWagnerUBaratsJOlbrichtSMousseauMNitzUMedenHfor the AGO-GINECO Intergroup2001 Epirubicin/Paclitaxel/Carboplatin (TEC) *vs*. Paclitaxel/Carboplatin (TC) in first line treatment of ovarian cancer FIGo stages IIb-IV. Interim results of an AGO-GINECO Intergroup phase III trial Proc Am Soc Clin Oncol 20 202a[Abstr]

[bib12] GICOG, (Gruppo Interregionale Cooperativo Oncologico Ginecologia), Italy1992 Long-term results of a randomized trial comparing cisplatin with cisplatin and cyclophosphamide with cisplatin, cyclophosphamide, and adriamycin in advanced ovarian cancer Gynecol Oncol 45 115117 1592277 10.1016/0090-8258(92)90272-k

[bib13] GregoryRKHillMEMooreJet al2000 Combining platinum, paclitaxel and anthracycline in patients with advanced gynaecological malignancy Eur J Cancer 36 503507 10717527 10.1016/s0959-8049(99)00309-3

[bib14] HillMMacfarlaneVMooreJet al1997 Taxane/platinum/anthracycline combination therapy in advanced epithelial ovarian cancer Semin Oncol 24Suppl 23437

[bib15] HudisCASeidmanADBaselgaJet al1995 Sequential adjuvant therapy with doxorubicin/paclitaxel/cyclophosphamide for resectable breast cancer involving four or more axillary nodes Semin Oncol 22 1823 8643965

[bib16] KayeSBPiccartMJAaproMet al1997 Phase II trials of docetaxel (Taxotere) in advanced ovarian cancer — an updated overview Eur J Cancer 33 21672170 9470802 10.1016/s0959-8049(97)00363-8

[bib17] MarkmanMKennedyAWebsterKet al2001 Combination chemotherapy with carboplatin and docetaxel in the treatment of cancers of the ovary and fallopian tube and primary carcinoma of the peritoneum J Clin Oncol 19 19011905 11283121 10.1200/JCO.2001.19.7.1901

[bib18] McGuireWPHoskinsWJBradyMFet al1996 Cyclophosphamide and cisplatin compared with paclitaxel and cisplatin in patients with stage III and stage IV ovarian cancer N Engl J Med 334 16 7494563 10.1056/NEJM199601043340101

[bib19] MillerABHoogstratenSStaquetMWinklerM1981 Reporting results of cancer treatment Cancer 74 207214 10.1002/1097-0142(19810101)47:1<207::aid-cncr2820470134>3.0.co;2-6 7459811

[bib20] MuggiaFMHainsworthJDJeffersSet al1997 Phase II study of liposomal doxorubicin in refractory ovarian cancer: antitumor activity and toxicity modification by liposomal encapsulation J Clin Oncol 15 987993 9060537 10.1200/JCO.1997.15.3.987

[bib21] MuggiaFMBralyPSBradyMFet al2000 Phase III randomized study of cisplatin versus paclitaxel versus cisplatin and paclitaxel in patients with suboptimal stage III or IV ovarian cancer: a Gynecologic Oncology Group study J Clin Oncol 18 106115 10623700 10.1200/JCO.2000.18.1.106

[bib22] NeijtJPTen BokkelHuininkWWVan der BergMELet al1991 Long term survival in ovarian cancer Eur J Cancer 17 13671372 10.1016/0277-5379(91)90011-2 1835850

[bib23] NeijtJPHansenMHansenSWet al1997 Randomised phase III study in previously untreated epithelial ovarian cancer FIGO stage IIB, IIC, III, IV, comparing paclitaxel–cisplatin and paclitaxel–carboplatin Proc Am Soc Clin Oncol 16 1259[Abstr]

[bib24] OmuraGABundyBNBerekJSet al1989 Randomized trial of cyclophosphamide plus cisplatin with or without doxorubicin in ovarian carcinoma: a Gynecologic Oncology Group Study J Clin Oncol 7 457465 2926470 10.1200/JCO.1989.7.4.457

[bib25] OzolsRFBundyBNFowlerJet al1999 Randomized phase III study of cisplatin (CIS)/paclitaxel (PAC) versus carboplatin (CARBO)/PAC in optimal stage III epithelial ovarian cancer (OC): a Gynaecologic Oncology Group Trial (GOG 158) Proc Am Soc Clin Oncol 18 356a[Abstr 1373]

[bib26] PiccartMJBertelsenKJamesKet al2000 Randomized intergroup trial of cisplatin–paclitaxel versus cisplatin–cyclophosphamide in women with advanced epithelial ovarian cancer: three-year results J Natl Cancer Inst 92 699708 10793106 10.1093/jnci/92.9.699

[bib27] RustinGJNelstropAEMcCleanPet al1996 Defining response of ovarian carcinoma to initial chemotherapy according to serum CA125 J Clin Oncol 14 15451551 8622070 10.1200/JCO.1996.14.5.1545

[bib28] VaseyPAPaulJJunorEJet al1999 Docetaxel–cisplatin in combination as first-line chemotherapy for advanced epithelial ovarian cancer J Clin Oncol 17 20692080 10561260 10.1200/JCO.1999.17.7.2069

[bib29] VaseyPAAtkinsonRColemanRet al2001a Docetaxel–carboplatin as first line chemotherapy for epithelial ovarian cancer Br J Cancer 84 170178 11161372 10.1054/bjoc.2000.1572 PMC2363708

[bib30] VaseyPAon behalf of the Scottish Gynecologic Cancer Trials Group2001b Preliminary results of the SCOTROC trial: a phase III comparison of paclitaxel–carboplatin (PC) and docetaxel–carboplatin (DC) as first-line chemotherapy for stage Ic–IV epithelial ovarian cancer (EOC) Proc Am Soc Clin Oncol 20[Abstr 804]

[bib31] YoungRCPerezCAHoskinsWJ1993 Cancer of the ovaryInCancer: Principles and Practice of Oncology4th edn, DeVita VT, Hellman S, Rosenberg SA. (eds)Philadelphia: JB Lippincott Co

